# How Help May Be Given

**Published:** 1914-03-21

**Authors:** 


### HOW HELP MAY BE GIVEN.

The doctors' wives who may become members of the proposed Association may be able to have their shopping done for them without trouble to themselves and at the minimum of cost. All they need do is to make out a list of the articles, or to send the name of any one article they may require. They should be helped in this task by the advertising pages of The Hospital, a study of which, and especially those sections which relate to enterprises within the scope of the Doctors' Wives Association, should enable them to decide readily the class of article they prefer, together with its exact price and full particulars. If they do not find this information in the advertisement pages of one issue, let them consult those of previous weeks. Failing a successful search, the next step will be to write out a full description of the articles desired, and send a written order for them, accompanied by a cheque to cover the estimated cost of the goods in question, including the carriage, addressed to the Secretary of the D.W. Association in compliance with the rules, a copy of which would be sent out in due course. Any sum left over will be held at the disposal of the correspondent to be dealt with as she may desire. The duty the Association will undertake is to select the best articles, to purchase them on the best possible terms, to see that the doctor's wife gets full value for her money, and to have the goods forwarded to her without any avoidable delay. Again, should she decide to come to London in order to make her own selection, she would advise the Association in advance of her intention and requirements, and they will supply her with introductions which will enable her to do her own shopping on the best terms. This plan should save her time and her money, whilst it secures for her an exceptional market full of attraction and advantages, the nature of which should soon become apparent as one direct result of the doctors' wives co-operating together for their mutual advantage and security.

